# High Expression of MicroRNA-200a/b Indicates Potential Diagnostic and Prognostic Biomarkers in Epithelial Ovarian Cancer

**DOI:** 10.1155/2022/2751696

**Published:** 2022-03-25

**Authors:** Beilei Zhang, Yi Li, Yanhong Li, Hongxi Zhao, Ruifang An

**Affiliations:** ^1^Department of Obstetrics and Gynecology, The First Affiliated Hospital of Xi'an Jiaotong University, Xi'an, Shaanxi, China; ^2^Department of Obstetrics and Gynecology, Tangdu Hospital, Air Force Medical University, Xi'an, China

## Abstract

**Objective:**

To detect the expression levels of microRNA-200a/b (miR-200a/b) in tumor tissues and serum of patients with epithelial ovarian cancer (EOC) and to explore its clinical significance.

**Methods:**

A retrospective selection of 30 cases of benign ovarian disease or healthy physical examination (control group) and 55 cases of EOC patients. Real-time quantitative PCR was used to detect the expression level of miR-200a/b in tumor tissues and serum, and the miR-200a/b expresses relevance in the two types of samples were evaluated at the same time. Receiver operating characteristic curve (ROC) and Kaplan-Meier survival analysis were used to evaluate the diagnostic value of miR-200a/b expression and its influence on prognosis, respectively.

**Results:**

The serum and tissue miR-200a/b expression levels in EOC patients were higher than those in the control group (*P* < 0.001), and there was a significant positive correlation between serum and tissue miR-200a/b expression (*R*^2^ = 0.9419, *P* < 0.001 and *R*^2^ = 0.9605, *P* < 0.001). ROC analysis showed that the expression of serum miR-200a/b can distinguish EOC patients from the control group. In addition, there were significant differences in the TNM stage, tumor differentiation, and lymph node metastasis between the miR-200a/b high- and low-expression groups (*P* < 0.05). Kaplan-Meier survival analysis found that the overall survival and disease-free survival of patients with high miR-200a/b expression were shorter than those of patients with low miR-200a/b expression (*P* < 0.05).

**Conclusion:**

Upregulation of miR-200a/b expression is a common molecular event in EOC patients, and miR-200a/b can be used as a noninvasive biomarker for the diagnosis and prognosis of EOC.

## 1. Introduction

Ovarian cancer is one of the three most common female malignancies, with a fatality rate ranking first among female malignancies and a 5-year survival rate of about 48% [1]. Epithelial ovarian cancer (EOC) accounts for 86%-91% of ovarian malignant tumors. It is more common in women aged 55-65. It has the characteristics of insidious early symptoms, prone to multiple organ implants and lymph node metastasis [2]. EOC patients have no obvious symptoms in the early stage of onset, and most patients are already at an advanced stage when they are diagnosed, so the prognosis is very poor. At present, carbohydrate antigen 125 (CA125) is the most commonly used biomarker to diagnose EOC, but its sensitivity for early diagnosis is only 40% [3]. Therefore, discovering new EOC diagnosis and prognostic biomarkers has very important clinical significance for enhancing the sensitivity and specificity of early diagnosis, optimizing treatment targets, and improving prognosis.

MicroRNA (miRNA) is a noncoding RNA with a length of 18-25 nucleotides, which can complementally bind to the 3′ untranslated region (UTR) of the target messenger RNA (mRNA), participating in the process of regulating gene transcription and translation, thereby regulating gene expression [4]. Over the years, with the continuous in-depth research on miRNA, scholars have discovered that miRNA is involved in regulating the posttranscriptional expression regulation of more than 30% of human genes [5]. It is not only related to cell development, differentiation, metabolism, aging, defense, and other life activities, but also has a close relationship with tumors [6]. Studies have shown that the occurrence and development of tumors are closely related to the abnormal expression of miRNAs in tissues, which play the role of protooncogene or tumor suppressor gene [7]. Due to the stable nature of miRNA and the small difference in the expression profile of miRNA in serum and plasma, miRNA may be used as a potential biomarker for cancer diagnosis and therapy [8].

Studies have found that the miR-200 family is abnormally expressed in EOC and a variety of malignant tumors [9, 10]. According to the similarity of miRNA seed sequences, members of the miR-200 family can be divided into two categories [11]. The first category includes miR-200b, miR-200c, and miR-429, which contain the same seed sequence (5′-AAUACUG-3′). The second category containing miR-200a and miR-141 shares another identical seed sequence (5′-AACACUG-3′). The two types of seed sequences differ by only one base. However, there are few studies on the expression of miR-200a/b in serum of EOC patients and tumor tissues. This study intends to detect the expression of miR-200a/b in serum and tumor tissues of EOC patients and analyze its clinical significance for EOC.

## 2. Materials and Methods

### 2.1. Ethics Statement Differentiation

This study was approved by the Ethics Committee of Air Force Medical University Tangdu Hospital, and written informed consent was obtained from each participant. All specimens were handled and made anonymous according to the ethical and legal standards.

### 2.2. Patients and Clinical Samples

The study subjects included 55 patients with EOC and 30 patients with benign ovarian diseases or healthy physical examinations, all of whom were treated at Tangdu Hospital of Air Force Medical University from January 2016 to December 2020. The age of EOC patients is 32-78 years (median age is 48 years); those with benign ovarian disease or health examination are 28-76 years old (median age is 46 years). The selection criteria for EOC patients were as follows: (1) pathologically confirmed patients with EOC by two different pathologists; (2) received no pre- or postoperative treatment such as chemotherapy or radiotherapy; (3) the patients had no previous history of other cancers; (4) the patients were provided with the detailed follow-up and clinical data. Paraffin blocks of ovarian tissues were obtained from EOC patients who have not undergone chemotherapy or patients with benign ovarian diseases. The control tissue and serum samples are from the benign ovarian diseases or healthy patients. And the EOC tissue and serum samples were taken from the EOC patients.

### 2.3. RNA Isolation and Quantitative Reverse Transcription PCR

The miRNeasy RNA purification kit (Catalogue No. 74004) was purchased from Qiagen, USA, the miRNA extraction and isolation kit (Catalogue No. 4992860) was purchased from Beijing Tiangen Co., Ltd., the SYBR PrimeScript RT-PCR kit (Catalogue No. RR014B) was purchased from Takara, Japan, and the 7900 QPCR instrument was purchased from ABI, USA. Serum miRNA extraction followed the steps: taking 5 ml of blood sample and centrifuging at 3000 r/min for 10 min. Then, take 200 *μ*l of serum, add 5 times the volume of QiAzol lysis Reagent, shake and mix, let stand for 5 min and add 3.5 *μ*l miRNeasy Serum/Plasma Spike-In Control, shake, and mix well. The remaining steps are carried out in accordance with the miRNeasy serum/plasma RNA purification kit (Catalogue No. 74004) instructions. Tissue miRNA extraction followed the steps: take 50-100 mg of tissue sample and follow the miRcute miRNA extraction and isolation kit instructions for miRNA extraction. The amount of extracted miRNA is stored at -80 degrees. Reverse transcription was performed using The PrimeScript RT-PCR reagent kit (Takara, Dalian, Catalogue No. RR014B, China). PCR amplification was carried out using SYBR Premix Ex TaqTM II (Takara) with ABI PRISM 7900 System (Applied Biosystems). The upstream primers are 5′-TAA CAC TGT CTG GTA ACG ATG T-3′ (miR-200a) and 5′-TAA TAC TGT CTG GTA ACG ATG T-3′ (miR-200b); the downstream primers are the primers provided in the kit. The upstream primer of the internal reference gene U6 is 5′-GTG CTC CCT GCT TCG GCA GCA CAT ATA C-3′, and downstream primer is 5′-AAA AAT ATG GAA CGC TTC ACG AAT TTG-3′. The expression level of miR-200a/b was analyzed by the relative quantitative method of 2^-△△CT^. Each qRT-PCR experiment was repeated in triplicate for each sample.

### 2.4. Statistical Analysis

Schapiro-Wilk test was used to check the normality distribution of the data before performing statistical analysis. Statistical analysis was performed with the GraphPad Prism 8.0 (GraphPad Software, San Diego, CA, USA). The measurement data was expressed by the mean standard deviation. The two-sample Student's *t*-test was used for the pairwise comparison. The *χ*^2^ test was used for the count data comparison. Pearson and Spearman's test was used for correlations analysis. The Kaplan-Meier method was used for the survival analysis, and the log-rank test was performed. The receiver operating characteristic curve (ROC) and the area under the ROC curve (AUC) were used to evaluate the diagnostic significance of miRNA. *P* < 0.05 was considered to indicate a statistically significant difference.

## 3. Results

### 3.1. miR-200a/b Expression in EOC Patients and Controls

**Figure 1:**
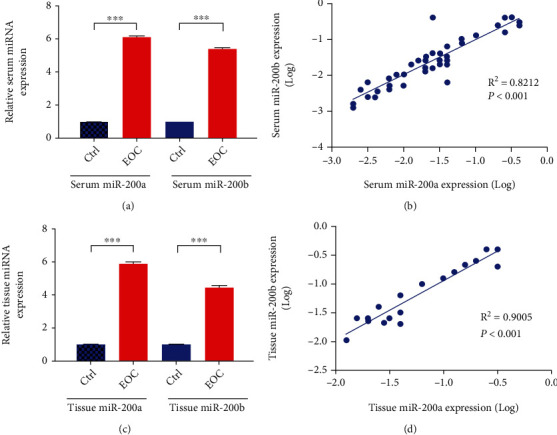
miR-200a/b expression in serum and tumor tissues of EOC patients. (a) The expression level of serum miR-200a/b in healthy controls and EOC patients. (b) The correlation between miR-200a and miR-200b expression in serum. (c) The expression level of miR-200a/b in tumor tissues of healthy controls and EOC patients. (d) The correlation between miR-200a and miR-200b expression in tumor tissues.

The qPCR was performed to detect serum miR-200a/b expression levels in all participants. QPCR results showed that serum miR-200a and miR-200b levels in EOC patients are significantly higher than the control group (6-fold and 5.4-fold, respectively) (*P* < 0.001, Figure 1(a)), and these two are significantly positively correlated (*R*^2^ = 0.8212, *P* < 0.001, Figure 1(b)). In addition, the level of miR-200a/b in tumor tissues of EOC patients was also significantly higher than that of the control group (*P* < 0.001, Figure 1(c)), and the two were also significantly positively correlated (*R*^2^ = 0.9005, *P* < 0.001, Figure 1(d)).

### 3.2. The Correlation of miR-200a/b Expression between Serum and Tumor Tissues

**Figure 2:**
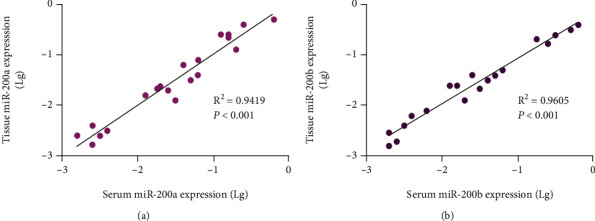
The correlation of miR-200a/b expression between serum and tumor tissues. (a) miR-200a; (b) miR-200b.

Spearman correlation analysis found that there was a significant positive correlation between the expression of miR-200a in serum and tumor tissues (*R*^2^ = 0.9419, *P* < 0.001, Figure 2(a)). Similarly, the expression level of miR-200b was also positively correlated in the two groups of different serum and tissues (*R*^2^ = 9605, *P* < 0.001, Figure 2(b)).

### 3.3. The Diagnostic Value of miR-200a/b for EOC

**Figure 3:**
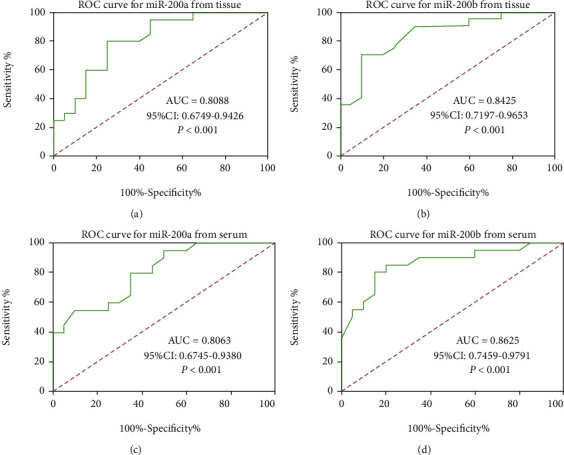
The diagnostic value of miR-200a/b in (a, b) tumor tissues and (c, d) serum of EOC patients. (a) miR-200a from tissue; (b) miR-200b from tissue; (c) miR-200a from serum; (d) miR-200b from serum.

We then investigated the diagnostic value of miR-200a/b from tissue or serum. ROC analysis showed that the expression of miR-200a/b both from tissue (Figures 3(a) and 3(b)) and serum (Figures 3(c) and 3(d)) can distinguish EOC patients from the control group, and the AUC was 0.8088 (95% CI: 0.6749~0.9426, Figure 3(a)), 0.8425 (95% CI: 0.7197~0.9653, Figure 3(b)), 0.8063 (95% CI: 0.6745~0.9380, Figure 3(c)), and 0.8625 (95% CI: 0.7459~0.9791, Figure 3(d)), respectively. These results suggest that the expression of miR-200a/b may be a potential molecular marker for distinguishing EOC from patients with benign ovarian diseases or healthy control.

### 3.4. The Relationship between miR-200a/b Expression and Clinical Parameters of EOC Patients

Using the Youden's index of 0.55 and 0.60 for miR-200a and miR-200b, respectively, EOC patients were divided into miR-200a/b high-expression and low-expression groups, and the differences in clinical parameters between the two groups were compared. As shown in Tables 1 and 2, there are significant differences in TNM stage, tumor differentiation, and lymph node metastasis between the miR-200a/b high- and low-expression groups (*P* < 0.05). The patient ratio of TNM stages III and IV in the miR-200a/b high-expression group was significantly higher than that in the miR-200a/b low-expression group (*P* = 0.006 and 0.003). And the ratio of well tumor differentiation in the miR-200a/b high-expression group was also higher than that in the miR-200a/b low-expression group (*P* = 0.003and*P* = 0.008). In addition, the incidence of lymph node metastasis in patients with low miR-200a/b expression was significantly lower than that in patients with high miR-200a/b expression (*P* = 0.021 and 0.013), and the incidence of ascites in patients with low miR-200a/b expression was also significantly lower (*P* = 0.014 and 0.026).

### 3.5. Prognostic Value of miR-200a/b Expression in EOC Patients

**Figure 4:**
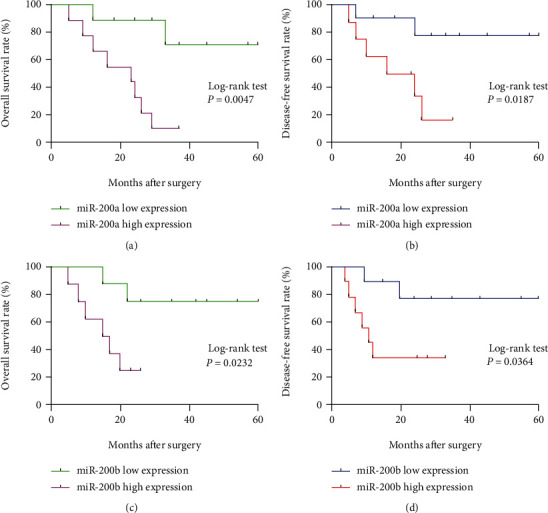
Kaplan-Meier survival curves of the EOC patients. (a) Effect of miR-200a expression on OS in EOC patients. (b) Effect of miR-200a expression on DFS in EOC patients. (c) Effect of miR-200b expression on OS in EOC patients. (d) Effect of miR-200b expression on DFS in EOC patients.

To assess the effect of miR-200a/b expression level on prognosis, patients were stratified into low and high miR-200a/b expression groups according to their median miR-200a/b expression levels. Kaplan-Meier survival analysis found that overall survival (OS) and disease-free survival (DFS) of patients with miR-200a high expression were significantly shortened (*P* = 0.0047 and *P* = 0.0187) (Figures 4(a) and 4(b)). Similarly, the OS and DFS of patients with miR-200b high expression were also statistically different from those with miR-200b low expression (*P* = 0.0232 and *P* = 0.0364) (Figures 4(c) and 4(d)). These results suggest that miR-200a/b might serve as a promising prognostic biomarker for EOC.

## 4. Discussion

Ovarian cancer is the malignant tumor with the highest mortality rate in women, and its pathogenesis is complicated. EOC is the most common pathological type among them. Due to the difficulty of early diagnosis, the high recurrence rate after surgical treatment, and the lack of specific ideal therapeutic drugs, most EOC patients relapse due to tumor cell proliferation and metastasis, which eventually leads to death [12]. Therefore, it is of great significance to actively seek effective biomarkers for the diagnosis and prognosis of ovarian cancer.

miRNA is a newly discovered noncoding small molecule RNA that participates in the regulation of cell growth and differentiation, energy metabolism, apoptosis, and other physiological processes. In recent years, it has been found that most miRNAs are located on chromosomal sites related to tumors. miRNAs can regulate tumor-related genes, participate in tumor cell proliferation, apoptosis, invasion, or angiogenesis, and are closely related to the occurrence and development of human tumors [13, 14]. miR-200 families are abnormally expressed in EOC and a variety of malignant tumors. Iorio et al. [15] screened a variety of miRNAs for EOC and found that the miR-200 family showed the highest fold of upregulation in EOC, suggesting that the miR-200 family plays an important role in the pathogenesis of EOC and can be used for the early diagnosis of EOC. Gong et al. [16] showed that in hepatocellular carcinoma (HCC) cells, miRNA-200a-3p can directly bind to its downstream target gene cyclin-dependent kinase 6 (CDK6) to inhibit the expression of CDK6 and inhibits the entry of HCC cell cycle into S phase, thereby inhibiting the proliferation of HCC. In addition, miRNA-200a can induce HCC apoptosis by targeting SIRT1 to promote the synthesis and secretion of apoptosis-related proteins [17]. Kurata et al. [18] found that the downregulation of miR-200c primarily regulated gastric cancer cell morphology by targeting E-cadherin through upregulation of ZEB1, thus resulting in poorly differentiated histology in gastric cancer. In addition, by detecting the expression of microRNA in glioma tissues, it was found that the expression of miRNA-200b was downregulated in glioma tissues, indicating that microRNA-200b is closely related to the prognosis of glioma, which can provide a basis for judging the prognosis of glioma patients [19]. Our study found that the expression of miR-200a/b in serum or tumor tissues was significantly upregulated compared with the control group. Because of their high homology, the expression of these two miRNAs showed a significant positive correlation. In the comparison of clinical parameters, we found that the high expression of miR-200a/b is associated with high-grade tumor stage, high differentiation, lymph node metastasis, and ascites and has nothing to do with patient age, tumor size, and plasma CA125 level. These results indicate that the biological function of miR-200a/b is highly related to tumor invasion and metastasis.

miR-200a belongs to the miR-200 family and is located on chromosome 1(1p33.36). It is paired with the 3′ untranslated region (3′UTR) of DCAMKL-1 target gene mRNA through the 5′ seed sequence and is responsible for regulating epithelial-mesenchymal transition [20]. Sanchez-Cid et al. showed that the expression of miR-200 family was significantly higher in patients with ductal breast cancer from tumors to distant metastases, suggesting that the level of miR-200s may be related to the growth and metastatic properties of breast luminal progenitor cells [21]. Pendlebury et al. showed that the high level of serum miR-200a is closely related to the tumor malignancy of patients with EOC and can be used as a biomarker for early detection of EOC [22]. In this study, the ROC curve showed that the area under the curve of serum miR-200a for the early diagnosis of EOC was 0.8088, and the sensitivity and specificity were both higher than 80%, suggesting that plasma miR-200a is a potential biomarker and has a certain effect on the screening and diagnosis of EOC. miR-200b and miR-200a belong to the same miR-200 cluster, and both map to chromosome 1(1p33.36). Many studies have reported abnormal expression of miRNA-200b in ovarian cancer. Kan et al. found that the expression level of miRNA-200b in EOC tissues was significantly higher than that in normal ovarian tissues [23]. Duong et al. reported that the high expression of miR-200 family was correlated to poor DFS (95% CI: 0.95-2.56) and poor OS (95% CI: 1.03-2.52) in breast cancer patients while downregulation of miRNA-200s was associated with poor OS (95% CI: 0.46-1.63) in triple-negative breast cancer (TNBC) patients and poor OS (95% CI: 0.27-0.88) in luminal breast cancer patient [24]. Several studies have shown increased tissue expression levels of miR-200a in late stages of OC (III and IV) [25–27]. Conversely, several other reports have demonstrated that the expression of miR-200a decreases with disease progression [28]. Meng et al. pointed out that the level of miRNA-200b in patients with ovarian cancer is highly expressed, and its expression level is closely related to CA125 [29].

However, our study did not find that the level of plasma miRNA-200b in EOC patients is related to the level of CA125. We found that high expression of miR-200b is associated with TNM stage, tumor differentiation, lymph node metastasis, and the incidence of ascites. Through ROC curve analysis, miR-200b expression is helpful to the diagnosis of EOC, with good specificity and sensitivity. Further survival analysis found that the high expression of miR-200a/b was associated with a significant reduction in OS and DFS in EOC patients, indicating that plasma miRNA-200a/b high levels have good EOC diagnostic and prognostic value and can be used as molecular markers for early diagnosis and prognostic prediction of EOC patients. Consistent with our results, it was found that miR-200a, miR-200b, and miR-200c were overexpressed in women with poor OS [30]. Meanwhile, high expression of miR-200a seems to be typical for overall and relapse-free survival [30, 31]. In addition, patients with low expression of miR-200a in tissues showed shorter relapse-free survival [32] and lower OS [28, 32]. In contrast, other groups reported an increase [33, 34] or a decrease [25, 35] expression of miR-200a, but the correlation between miRNA levels and patient survival was not investigated. Finally, some reports did not find that there existed a relationship between miR-200a expression levels and patient OS [23, 33]. Overall, these findings suggest that, in most cases, the expression pattern of miR-200a is indicative of an oncogenic process and may prove clinically valuable for patient prognosis. More efforts are needed to investigate the role of miRNA-200a/b in EOC.

In conclusion, upregulation of miRNA-200a/b expression is a common molecular event in EOC patients, and serum miR-200a/b might serve as a reliable and noninvasive biomarker for the early diagnosis and prognosis prediction of EOC.

## Figures and Tables

**Table 1:** Correlation between miR-200a expression and clinical parameters in EOC patients.

Serum miR-200a expression				
Parameters	Number	Low (*n* = 25)	High (*n* = 30)	*P* value
*Age*				0.245
≥60	29	13	16	
<60	26	12	14	
*Tumor size*				0.176
≥8 cm	32	15	17	
<8 cm	23	10	13	
*TNM stage*				0.006
I/II	27	16	11	
III/IV	28	9	19	
*Tumor differentiation*				0.003
Well	25	4	21	
Moderate	13	7	6	
Poor	17	14	3	
*Lymph node metastasis*				0.021
Negative	23	16	7	
Positive	32	9	23	
*Ascitic fluid*				0.014
Negative	17	13	4	
Positive	38	12	26	
*CA125 level (u/ml)*				0.532
≥500	27	13	14	
<500	28	12	16	

**Table 2:** Correlation between miR-200b expression and clinical parameters in EOC patients.

Serum miR-200b expression				
Parameters	Number	Low (*n* = 25)	High (*n* = 30)	*P* value
*Age*				0.178
≥60	21	10	11	
<60	34	15	19	
*Tumor size*				0.236
≥8 cm	26	12	14	
<8 cm	29	13	16	
*TNM stage*				0.003
I/II	26	18	8	
III/IV	29	7	22	
*Tumor differentiation*				0.008
Well	24	5	19	
Moderate	12	6	6	
Poor	19	14	5	
*Lymph node metastasis*				0.013
Negative	24	19	5	
Positive	31	6	25	
*Ascitic fluid*				0.026
Negative	25	18	7	
Positive	30	7	23	
*CA125 level (u/ml)*				0.362
≥500	21	9	12	
<500	34	16	18	

## Data Availability

The data is available on request.
